# Dietary Patterns and Fitness Level in Mexican Teenagers

**DOI:** 10.1155/2018/7159216

**Published:** 2018-05-02

**Authors:** César Estrada-Reyes, Patricia Tlatempa-Sotelo, Roxana Valdés-Ramos, María Cabañas-Armesilla, Rafael Manjarrez-Montes-de-Oca

**Affiliations:** ^1^Centro de Investigación y Estudios Avanzados en Ciencias de la Salud, Universidad Autónoma del Estado de México, Toluca, MEX, Mexico; ^2^Universidad Complutense de Madrid, Madrid, Spain; ^3^Facultad de Ciencias de la Conducta, Licenciatura en Cultura Física y Deporte, Universidad Autónoma del Estado de México, Toluca, MEX, Mexico

## Abstract

**Background:**

Nowadays, the term “physical fitness” has evolved from sports performance to health status, and it has been considered a strong predictor of cardiovascular disease. In this sense, test batteries have been developed to evaluate physical fitness such as the ALPHA-FIT battery. On the other hand, the analysis of dietary patterns has emerged as an alternative method to study the relationship between diet and chronic noncommunicable diseases. However, the association between dietary patterns and the physical fitness level has not been evaluated in both adults and adolescents. This association is most important in adolescents due to the fact that establishing healthy dietary behaviors and a favorable nutritional profile in early stages of life prevents various chronic-degenerative diseases.

**Objective:**

To analyze the association between dietary patterns and the level of fitness in Mexican teenagers.

**Methods:**

We analyzed the relationship between dietary patterns and the fitness level of 42 teenage students in Toluca, Mexico. Students were weighed and measured, and their food intake was recorded for 2 weekdays and one weekend day. Dietary patterns were obtained by factorial analysis. The ALPHA-FIT battery was used to measure the fitness level.

**Results:**

Fifty percent of the students were found to have a low fitness level (62.1% men; 37.9% women). There was no association (*X*2 = 0.83) between the dietary patterns “high in fat and sugar,” “high in protein”, and “low in fat and protein” and the level of physical condition in teens.

**Conclusions:**

In this study, all of teenagers with a very low level of fitness obtained a high dietary pattern in protein; however, 40% with a high level of physical condition resulted in the same pattern; that is why we did not find a relationship between the fitness level and the patterns investigated in this study.

## 1. Introduction

The concept of physical fitness has evolved from its traditional orientation, linked to sports performance, to a much closer orientation related to health [1].

Physical fitness constitutes an integrated measure of all the functions and structures involved in the performance of physical activity or exercise [2]; also, a low fitness index is considered a strong predictor of cardiovascular disease [3], mainly cardiorespiratory fitness.

The fitness level can be assessed objectively through laboratory and field testing. Field tests are a good alternative to laboratory tests because of their easy execution, low cost, absence of sophisticated technical equipment, and time to perform them. Consequently, many adolescents may be evaluated simultaneously [4].

The objective of the ALPHA-FIT (Assessing Levels of Physical Activity and Fitness) study was to propose a battery of instruments to assess physical activity and fitness in a comparable way in different countries [4]. The ALPHA-FIT battery includes the following tests [5]: (1) 20-meter round-trip test to evaluate the aerobic capacity (Course Navette), (2) manual holding force test (dynamometry), (3) long jump test with feet together to assess the musculoskeletal ability (horizontal jump), (4) 10-meter round-trip test (4 × 10), and (5) body mass index (BMI).

The analysis of dietary patterns has emerged as an alternative method to study the relationship between diet and chronic noncommunicable diseases [6]. In this type of analysis, nutrients or foods are not analyzed in isolation, but food is combined into one or more composite variables, which allows a more complete view of the diet as a whole and its influence on health [6, 7]. In this way, the identification and study of dietary patterns may help to understand the relationship between diet and health [5].

The relationship between dietary patterns and disease risk has been demonstrated in adults, but not adolescents, despite the widespread recognition of the importance of establishing healthy dietary behaviors and a favorable nutritional profile in early stages of life to prevent various chronic-degenerative diseases [8].

The relationship between dietary patterns and cardiorespiratory fitness has been demonstrated in several studies [3, 9–12]. However, cardiorespiratory fitness is only a fragment of physical fitness which included other components [5]. To our knowledge, there are no studies which examine the relationship between both dietary patterns and the fitness level in adolescents; then, the purpose of this study was to analyze the association between dietary patterns and the fitness level of Mexican teenagers.

## 2. Materials and Methods

A cross-sectional study was carried out at “Nezahualcóyotl” high school in Toluca City, Mexico. We worked with 56 adolescent students between 14 and 17 years of age, by convenience sampling.

An informed assent letter was given to students wishing to participate, and another letter of informed consent was given to be signed by their parents.

This research was approved by the ethics committee of the Center for Research in Medical Sciences (CICMED) of the Autonomous University of the State of Mexico.

Weight was measured with a Tanita® BF-680W scale, while stature was measured with a Seca 213 portable stadiometer. With the above data, body mass index (BMI) was calculated and classified as very low, medium, high, and very high, following the classification of the ALPHA-FIT battery.

Subsequently, they were given 3 food registration formats of 24 hours each, to be filled for three days, of which one was on the weekend.

Next, we carried out the field tests as follows: Course Navette, dynamometry, horizontal jump, and speed 4 × 10 m. The results of each test were classified as very low, low, medium, high, and very high. The four tests were performed following the procedures of the *ALPHA-Fitness Battery Manual: Field Test for the Evaluation of Health-Related Physical Condition in Children and Adolescents* [13].

The 24-hour food records were collected at the school, after being filled out by the adolescents in their homes. The information obtained from these records was captured in a database in Microsoft Excel 2016 and later analyzed through the software Nutrimind®, version 11.0. We used a principal components factor analysis (PCFA), with varimax rotation with Kaiser normalization, examining the correlation matrix (rotated to facilitate its interpretability) among food consumption variables and reducing it to a smaller set of dimensions. These are factors (patterns), which capture the main characteristics of the diet in the studied population.

In the present study, the food groups defined for the construction of these standards were fatty vegetables, fruits, cereals, and tubers without fat (CATWOF), cereals and tubers with fat (CATWF), legumes (LEG), very low-fat animal products (VLFAP), low-fat animal products (LFAP), moderate-fat animal products (MFAP), high-fat animal products (HFAP), semiskimmed milk (SSM), whole milk (WM), milk with sugar (MWS), oils and fats without protein (OAFWOP), oils and fats with protein (OAFWP), sugar without fat (SWOF), and sugar with fat (SWF).

The correlation matrix was evaluated by the Bartlett sphericity test and the Kaiser–Meyer–Olkin (KMO) measure of sample adequacy. In the proposed analysis, the number of eating patterns (retained factors) was defined based on the following criteria: obtaining eigenvalue greater than 1.5 and factorial interpretability.

The denomination of each pattern was based on the food groups that were dominant in the analysis, for which the presence of absolute factor load ≥0.60 was established as a criterion.

To group the subjects into a given factor, we analyzed the scores obtained from the analysis so that the highest positive score or nearest to zero (when all scores were negative) was selected to identify the pattern to which the study subjects belonged.

Finally, we identified the association of dietary patterns with the physical fitness level of adolescents, through the chi-square statistical test. A *p* value less than 0.05 was considered statistically significant. All statistical analyses were performed with SPSS software version 23.

## 3. Results

The physical fitness level of 42 students was analyzed, of which 29 were female (69%) and 13 were male (31%), with a minimum age of 14 years and a maximum age of 17 years, presenting an average age of 15.6 years. BMI was classified as very low, low, medium, high, and very high, giving a percentage of 2.4, 2.4, 52.6, 28.6, and 14.3, respectively. The above data are presented in Table 1.

The results of the 4 field tests for fitness assessment were classified as very low, low, medium, high, and very high. The tests with the highest number of very low results for the total population were those of dynamometry and the horizontal jump with 57.1 and 52.4%, respectively. In contrast, the test that obtained the highest percentage of very high results was the speed test 4 × 10 m, with a 7.1%. The results of the tests are shown in Table 2.

Each test was assigned a certain value to obtain an average and thus to identify the overall fitness level of the students. Of the total number of students evaluated, 50% had a low level of fitness, with women predominating at this level (62.1%). It should be noted that no participant was able to obtain a very high rating on their overall fitness level and no woman had a very low level of fitness. The results of this are shown in Table 3.

On the other hand, we analyzed the level of overall physical condition according to the BMI that the adolescents presented. At the very low and low overall physical fitness levels, students with a very high and high BMI predominate. The results are shown in Table 4.

In the multidimensional characterization of the feed using PCFA, a KMO of 0.77 and a Bartlett sphericity of 0.035 were obtained as measures of sample adequacy. This indicates that the analysis carried out by virtue of the extension of the sample is justified.

The PCFA revealed three factors: pattern 1 explained 13.7% of the variability, which showed a stronger factor load in the vegetable group, OAFWP, and SWF. Pattern 2 accounted for 27.1% and was characterized by higher consumption of LFAP, HFAP, and OAFWP. Pattern 3 accounted for 39.3% and composed only of VLFAP. The three factors explained 80.1% of the total variance in consumption of the 16 food groups.  Table 5 shows the load factor rotated for the three dietary patterns identified and the name assigned for each pattern. A positive charge indicates positive association with the factor. The highest burden of a food group is the highest contribution of the food group to a specific factor or a pattern of food.

Later, the relationship between dietary patterns and the overall fitness level was analyzed, obtaining related results among them. Forty percent of adolescents who had a high fitness level were found in both patterns 1 and 2, and the only person with a very low fitness level was in pattern 2. The data above are shown in Table 6.

Finally, the chi-square statistical test was performed, which showed a score of 0.83, indicating that there is no association between the dietary patterns obtained by adolescents and the level of physical condition present in them.

## 4. Discussion

The aim of our study was to analyze the association between dietary patterns and the level of fitness in Mexican teenagers.

In the present study, 3 dietary patterns were identified: high in fat and sugar, high in protein, and low in fat. Several university studies indicate that students tend to choose foods rich in fats and carbohydrates and low in dietary fiber [14–16]. A study by Duran et al. [17] showed a low consumption of fruits and vegetables, a situation similar to another study in Spain, where 84.9% did not reach the dietary recommendations [18], reaching only 4.7% of the suggested fruit intake. Finally, Rodríguez et al. [19] indicate that, in university students, the socioeconomic level is unrelated to eating habits.

The majority of students tested, regardless of sex, as well as in other studies [15–20], eat energy-rich, high-fat, high-sugar, and protein foods that contribute significantly to total energy intake.

The results of the present study show excessive consumption of fats and proteins and low intake of carbohydrates. With respect to the studies in Guipúzcoa [21] and Sweden [22], with adolescents of the same age, it was observed how protein intake is generally superior to all the other studies. With respect to the consumption of carbohydrates, the proportion is smaller than that reported by other studies, especially with respect to the study in Sweden [21–23].

Regarding the level of physical condition of the adolescents, 50% of the participants showed a low fitness level. These results are not in agreement with those found by Ortega et al. [24] in Spanish adolescents who participated in the AVENA study. Mota et al. [25] in adolescents showed higher percentages of subjects with a low fitness level than those obtained in our study.

Some studies [26–28] have revealed a progressive and alarming deterioration in the physical condition of adolescents compared to what had happened in previous decades, and this is attributed mainly to the increase of sedentary behavior in the industrialized societies [27]; this situation is similar to the results shown in this study.

With the passage of time, it has been detected that adolescents are both less physically active and present a greater sedentary behavior. Due to this energy imbalance, problems have been documented because of which institutions and organizations worldwide have been alerted. This impairs their health, causing pathologies such as overweight and obesity [29].

When linking results of the association between dietary patterns and the level of fitness in adolescents, with other studies, no information was found evaluating the same variables. Most of the studies are focused only on cardiorespiratory fitness. In our point of view, although the relationship between cardiorespiratory fitness and health [3, 9] or dietary patterns [10–12] is well known, it is important to know how other variables of physical fitness could help to explain this relation.

The main limitation of this study was the low participation of adolescents, which was due to our convenience sampling. Moreover, we should evaluate whether our method to assess both physical fitness and dietary patterns is adequate to upcoming studies, for example, to assess only cardiorespiratory fitness.

However, we thought that results shown in this study are interesting, and they open a valuable opportunity for other investigations.

## 5. Conclusions

Although our results are not conclusive, this research shows data not studied previously in a population of high-school students. In this way, it deepens the knowledge and constitutes valuable information to take effective measures of public health and health promotion based on the evidence of the obtained results, besides helping to prevent possible diseases in the future.

There is a considerable percentage of our population that presents low levels of physical fitness, which should not be neglected.

All the adolescents with a very low level of fitness obtained a high-protein dietary pattern; however, 40% with a high level of physical condition resulted in the same pattern; that is why we found no relationship between the fitness level and the patterns investigated in this study.

More studies are needed with the variables included in the present evaluation and larger sample size.

## Figures and Tables

**Table 1:** Age (years) and BMI (%) by sex and total number of adolescents in the study.

BMI (body mass index)	Men (*n*=13)	Women (*n*=29)	Total (*n*=42)
Age	15.41 ± 0.9	15.51 ± 1.0	15.6 ± 0.9
Very low	0.0 (0/13)	3.4 (1/29)	2.4 (1/42)
Low	7.7 (1/13)	0.0 (0/29)	2.4 (1/42)
Medium	76.9 (10/13)	41.4 (12/29)	52.4 (22/42)
High	0.0 (0/13)	41.4 (12/29)	28.6 (12/42)
Very high	15.4 (2/13)	13.8 (4/29)	14.3 (6/42)

**Table 2:** Results of physical fitness tests of adolescents (%).

Tests	Men (*n*=13)	Women (*n*=29)	Total (*n*=42)
*Course Navette*			
Very low	7.7	13.8	11.9
Low	30.8	58.6	50.0
Medium	23.1	13.8	16.7
High	38.5	10.3	19.0
Very high	0.0	3.4	2.4
*Dynamometry*			
Very low	46.2	62.1	57.1
Low	7.7	20.7	16.7
Medium	38.5	13.8	21.4
High	0.0	0.0	0.0
Very high	7.7	3.4	4.8
*Horizontal jump*			
Very low	53.8	51.7	52.4
Low	15.4	20.7	19.0
Medium	23.1	24.1	23.8
High	7.7	3.4	4.8
Very high	0.0	0.0	0.0
*Speed 4 × 10 m*			
Very low	30.8	31.0	31.0
Low	38.5	34.5	35.7
Medium	23.1	6.9	11.9
High	0.0	20.7	14.3
Very high	7.7	6.9	7.1

**Table 3:** Overall fitness level (%): global fitness level.

Global fitness level	Men (*n*=13)	Women (*n*=29)	Total (*n*=42)
Very low	7.7 (1/13)	0.0 (0/29)	2.4 (1/42)
Low	23.1 (3/13)	62.1 (18/29)	50.0 (21/42)
Medium	53.8 (7/13)	27.6 (8/29)	35.7 (15/42)
High	15.4 (2/13)	10.3 (3/29)	11.9 (5/42)
Very high	0.0 (0/13)	0.0 (0/29)	0.0 (0/42)

**Table 4:** Overall fitness level and its relation to BMI (%).

Global fitness level	Body mass index				
	Very low (*n*=2)	Low (*n*=2)	Medium (*n*=29)	High (*n*=15)	Very high (*n*=8)
Very low	0.0 (0/2)	0.0 (0/2)	0.0 (0/29)	0.0 (0/15)	16.7 (1/8)
Low	100 (2/2)	100 (2/2)	22.7 (7/29)	91.7 (14/15)	50.0 (4/8)
Medium	0.0 (0/2)	0.0 (0/2)	63.6 (18/29)	0.0 (0/15)	16.7 (2/8)
High	0.0 (0/2)	0.0 (0/2)	13.6 (5/29)	8.3 (1/15)	16.7 (1/8)
Very high	0.0 (0/2)	0.0 (0/2)	0.0 (0/29)	0.0 (0/15)	0.0 (0/8)

**Table 5:** Load factors according to the factorial analysis.

Food groups	Dietary patterns		
	High in fat and sugar	High in protein	Low in fat and protein
Vegetables	0.735	—^1^	—
Fruits	—	—	—
CATWOF	—	—	—
CATWF	—	—	—
LEG	—	—	—
VLFAP	—	—	0.602
LFAP	—	0.640	—
MFAP	—	—	—
HFAP	—	0.718	—
SSM	—	—	—
WM	—	—	—
MWS	—	—	—
OAFWOP	—	0.605	—
OAFWP	0.663	—	—
SWOF	—	—	—
SWF	0.608	—	—

^1^Values <0.60 were excluded to simplify.

**Table 6:** Relationship of dietary patterns and the fitness level (%).

	Very low (*n*=1)	Low (*n*=21)	Medium (*n*=15)	High (*n*=5)	*P*
Pattern 1	0.0	42.9	40.0	40.0	0.83
Pattern 2	100.0	28.6	26.7	40.0	0.83
Pattern 3	0.0	28.6	33.6	20.0	0.83
